# Supplementary figures and images for: Pseudomonas aeruginosa type IV minor pilins and PilY1 regulate virulence by modulating FimS-AlgR activity
Source: PLoS Pathog. 2018 May 18;14(5):e1007074. doi: 10.1371/journal.ppat.1007074 (PMC5979040; doi:10.1371/journal.ppat.1007074)

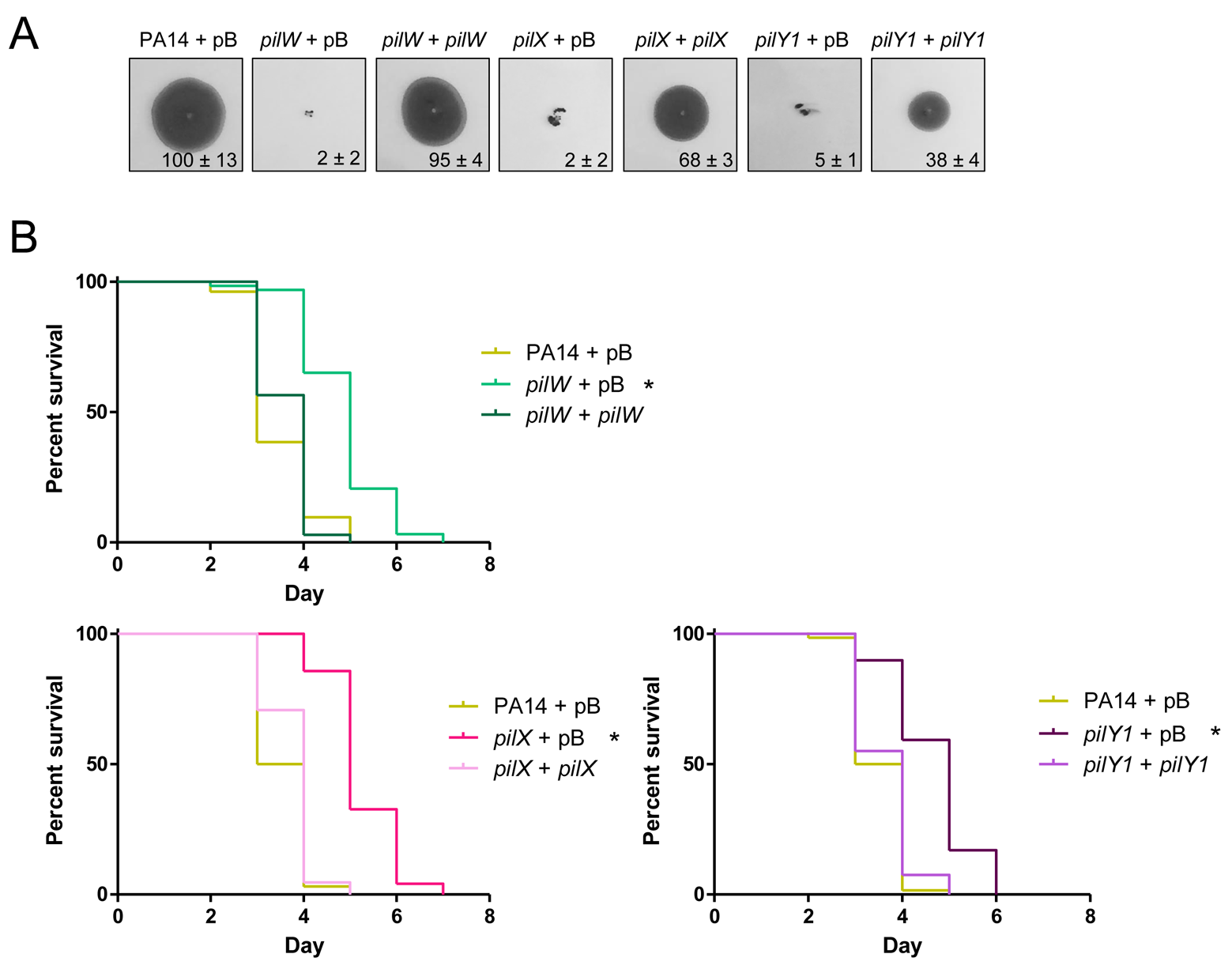

Supplement: S1 Fig — (A) Twitching motility assays for complemented PA14 pilW, pilX, and pilY1 mutants. Colonies were stab-inoculated into 1% agar LB plates, in triplicate. Plates were stained with crystal violet after 48 h at 30°C. Complementation of PA14 pilW, pilX, and pilY1 mutants with pBADGr-pilW, pBADGr-pilX, or pBADGr-pilY1, respectively, led to increased TM relative to complementation with pBADGr alone. Numbers indicate percent twitching area relative to WT, n = 3. (B) SK assays for complemented PA14 pilW, pilX, and pilY1 mutants. Complementation of pilW, pilX, and pilY1 mutants with pBADGr-pilW, pBADGr-pilX, or pBADGr-pilY1, respectively, restored virulence to near-WT levels. Asterisks indicate strains that were less virulent than PA14 + pBADGr by Gehan-Breslow-Wilcoxon test at p = 0.05 (p = 0.00833 with a Bonferroni correction), n = 3. Individual graphs represent separate trials. (TIF) [file ppat.1007074.s001.tif]

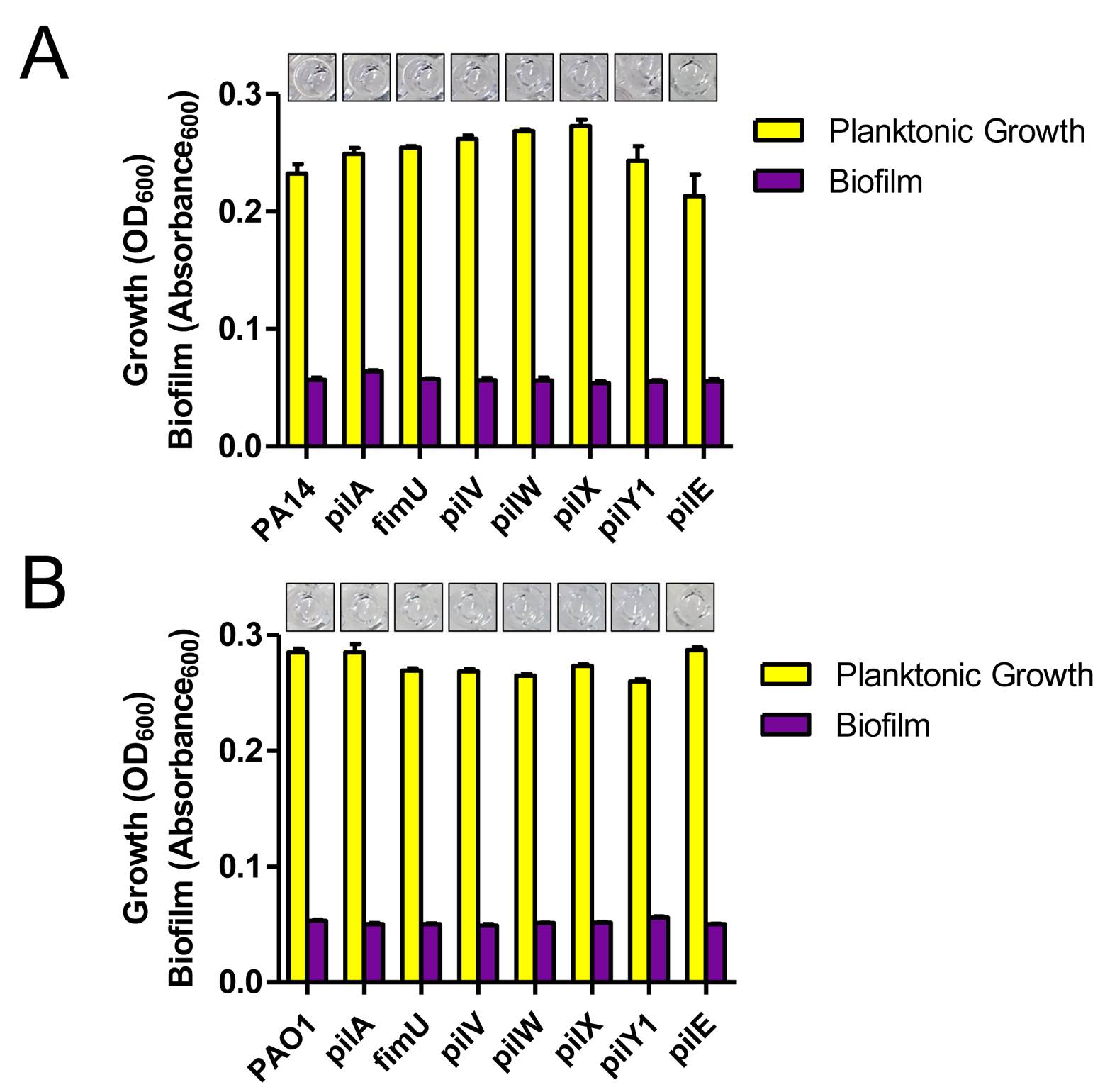

Supplement: S2 Fig — Biofilm assays for (A) PA14 and (B) PAO1 pilA, fimU, pilV, pilW, pilX, pilY1, and pilE mutants. Very little biofilm formation was detectable in liquid SK media for any strains. There were no differences in biofilm formation as determined by one-way ANOVA followed by Dunnett post-test relative to WT at p = 0.05, n = 3. (TIF) [file ppat.1007074.s002.tif]

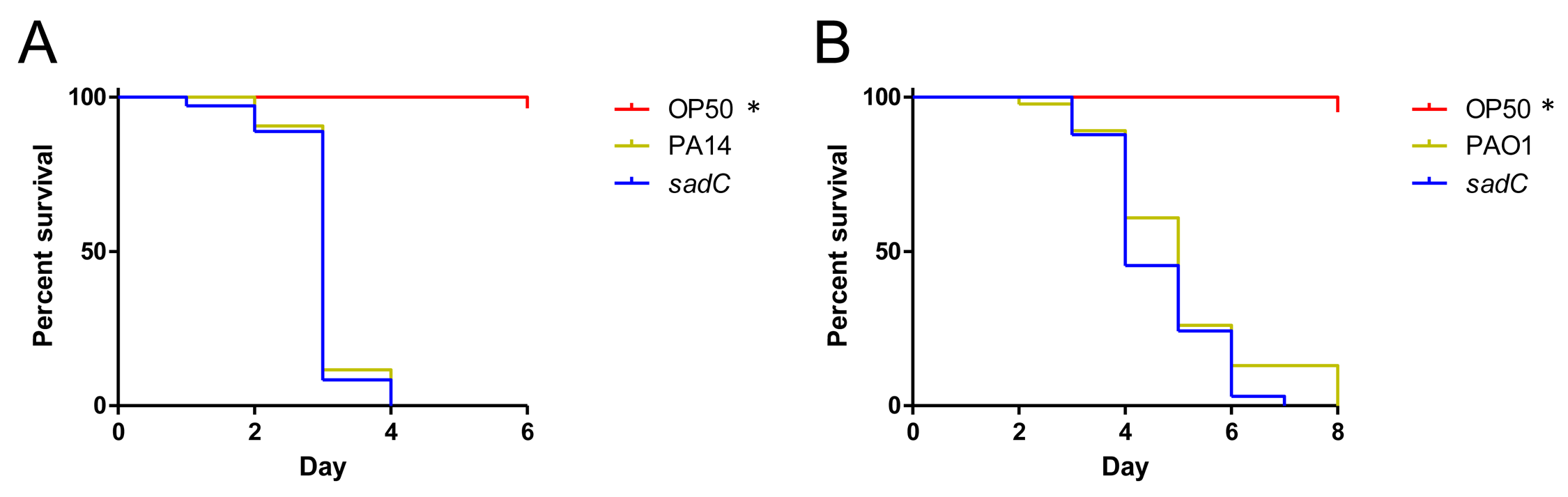

Supplement: S3 Fig — SK assays for (A) PA14 and (B) PAO1 sadC mutants. Loss of sadC had no impact on pathogenicity relative to each respective WT strain, as measured by Gehan-Breslow-Wilcoxon test at p = 0.05 (p = 0.025 with a Bonferroni correction), n = 3. (TIF) [file ppat.1007074.s003.tif]

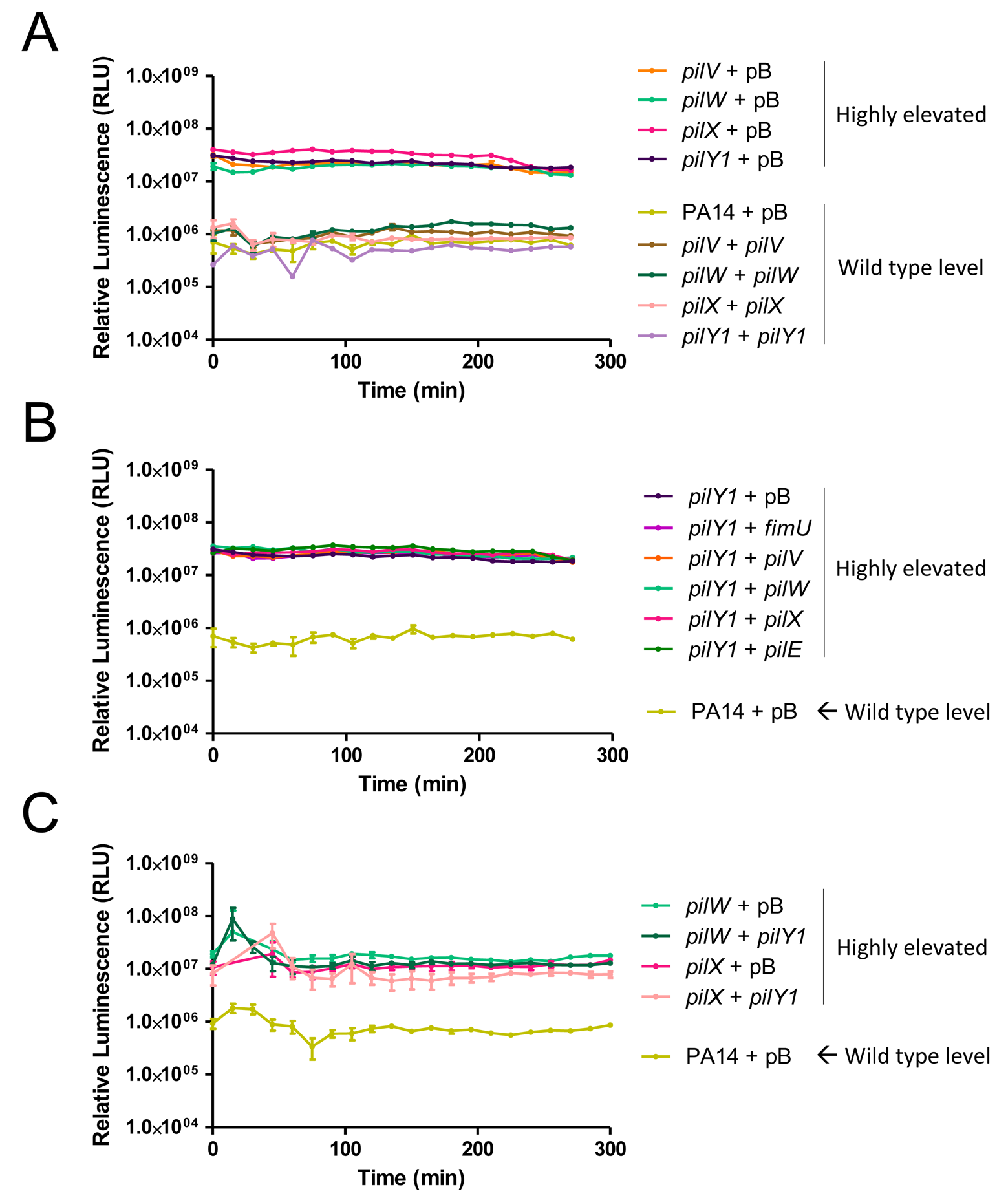

Supplement: S4 Fig — (A) fimU promoter activity of pilV, pilW, pilX, and pilY1 mutants complemented with the respective gene in trans. The high luminescence of each mutant was restored to WT level when pilV, pilW, pilX, and pilY1 were complemented with PilV, PilW, PilX, and PilY1, respectively. (B) fimU promoter activity of a pilY1 mutant expressing each MP in trans. Expression of FimU, PilV, PilW, PilX, or PilE in the pilY1 background had no impact on fimU promoter activity relative to the pilY1 + empty vector control. (C) fimU promoter activity of pilW and pilX mutants overexpressing PilY1. Overexpression of PilY1 had no impact on fimU promoter activity in pilW and pilX backgrounds relative to the respective vector-only controls. Assays in (A), (B), and (C) were carried out in the presence of 0.05% L-arabinose to induce expression of the pBADGr promoter, n = 3. (TIF) [file ppat.1007074.s004.tif]

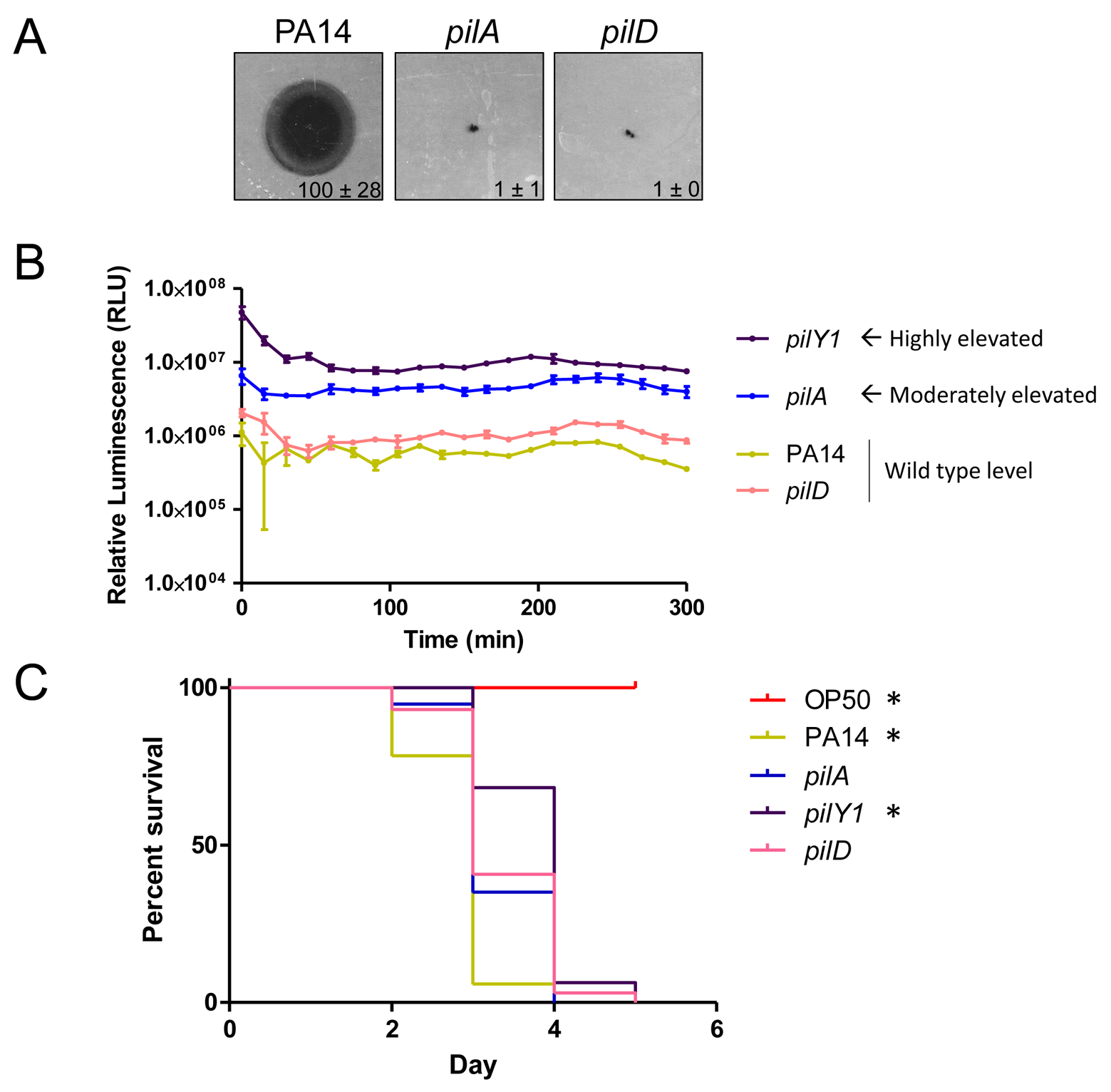

Supplement: S5 Fig — (A) Twitching motility assays for PA14 pilA and pilD mutants. Loss of pilD resulted in loss of twitching motility. Numbers indicate percent twitching area relative to WT, n = 3. (B) fimU promoter activity of a pilD mutant compared to PA14, pilA, and pilY1. Loss of pilD had no impact on fimU promoter activity relative to WT, n = 3. (C) SK assays for PA14, pilA, pilY1, and pilD mutants. A pilD mutant had equivalent virulence to a pilA mutant; less pathogenic than WT but more pathogenic than a pilY1 mutants. Asterisks represent strains that were significantly different from the pilA mutant by Gehan-Breslow-Wilcoxon test at p = 0.05 (p = 0.0125 with a Bonferroni correction), n = 3. (TIF) [file ppat.1007074.s005.tif]

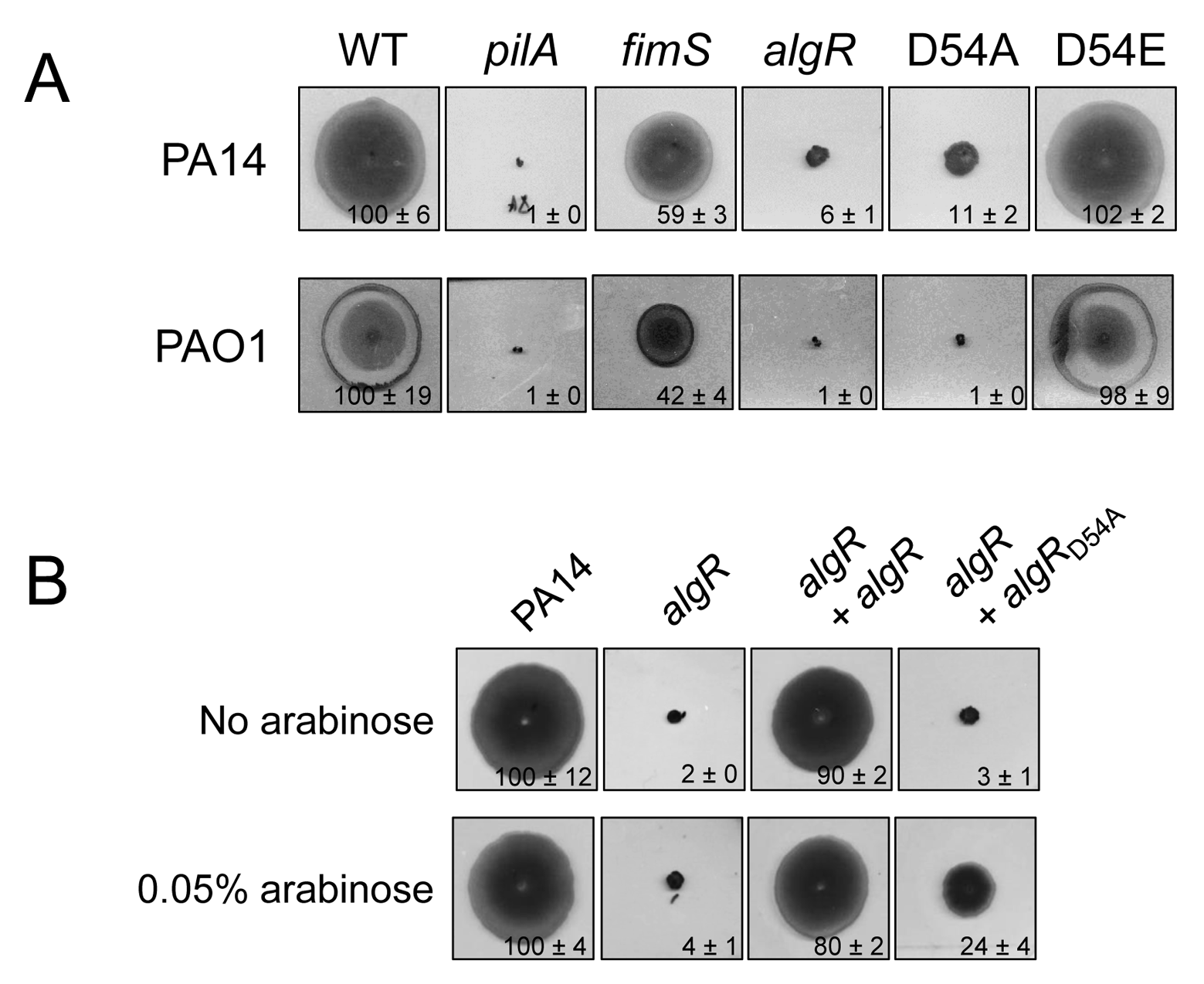

Supplement: S6 Fig — (A) Twitching motility assays for PA14 pilA, fimS, algR, algRD54A, and algRD54E mutants. Twitching motility was abolished in pilA, algR, and algRD54A mutants, and fully retained in the algRD54E mutant. A fimS mutant twitched to ~50% WT levels. (B) Twitching motility assays for PA14 algR complemented with AlgR or AlgRD54A. An algR mutant was fully complemented by AlgR with and without induction by 0.05% L-arabinose. The AlgRD54A variant supported twitching motility in the algR mutant background in the presence of 0.05% L-arabinose, to ~25% WT levels. In (A) and (B), numbers indicate percent twitching area relative to WT, n = 3. (TIF) [file ppat.1007074.s006.tif]
