# Supplementary material for: Pseudomonas aeruginosa type IV minor pilins and PilY1 regulate virulence by modulating FimS-AlgR activity
Source: PLoS Pathog. 2018 May 18;14(5):e1007074. doi: 10.1371/journal.ppat.1007074 (PMC5979040; doi:10.1371/journal.ppat.1007074)
Supplement: S1 Table — (DOCX) [file ppat.1007074.s007.docx]

**Table S1. Bacterial strains and plasmids used in this study.**

| **Strain/Plasmid** | **Characteristics** | **Source** |
| --- | --- | --- |
| **Plasmids** |  |  |
| pEX18Gm | Suicide vector for gene replacement | [1] |
| pEX18Gm-*pilA* | Deletion construct for PA14 *pilA* | This work |
| pEX18Gm-*fimU* | Deletion construct for PA14 *fimU* | This work |
| pEX18Gm-*pilV* | Deletion construct for PA14 *pilV* | This work |
| pEX18Gm-*pilW* | Deletion construct for PA14 *pilW* | This work |
| pEX18Gm-*pilX* | Deletion construct for PA14 *pilX* | This work |
| pEX18Gm-*pilY1* | Deletion construct for PA14 *pilY1* | This work |
| pEX18Gm-*pilE* | Deletion construct for PA14 *pilE* | This work |
| pEX18Gm-*sadC* | Deletion construct for *sadC* | This work |
| pEX18Gm­-*fimS* | Deletion construct for *fimS* | This work |
| pEX18Gm-*algR* | Deletion construct for *algR* | This work |
| pEX18Gm-*algR*_D54A_ | Mating construct for *algR* D54A substitution | This work |
| pEX18Gm-*algR*_D54E_ | Mating construct for *algR* D54E substitution | This work |
| pEX18Gm-*algU* | Deletion construct for *algU* | This work |
| pEX18Gm-*pilD* | Deletion construct for *pilD* | This work |
| pBADGr | Arabinose-inducible complementation vector | [2] |
| pBADGr-*pilW* | Complementation construct for *pilW* | [3] |
| pBADGr-*pilX* | Complementation construct for *pilX* | This work |
| pBADGr-*pilY1* | Complementation construct for *pilY1* | This work |
| pBADGr-*sadC* | Complementation construct for *sadC* | This work |
| pBADGr-*algR* | Complementation construct for *algR* | This work |
| pBADGr-*algR*_D54A_ | Complementation construct for *algR*_D54A_ | This work |
| pMS402 | Transcriptional reporter vector carrying the promoterless *luxCDABE* genes | [4] |
| pMS402-P*fimU* | Transcriptional reporter for *fimU* promoter | This work |
| pMS402-P*cdrA* | Transcriptional reporter for *cdrA* promoter | This work |
| pKT25 | Vector encoding T25 fragment of *B. pertussis* CyaA | [5] |
| pKT25-*fimS* | Vector encoding *fimS* fused to T25 | This work |
| pUT18C | Vector encoding T18 fragment of *B. pertussis* CyaA | [6] |
| pUT18C-*fimS* | Vector encoding *fimS* fused to T18 | [7] |
| pUT18C-*algR* | Vector encoding *algR* fused to T18 | This work |
| pUT18C-*pilA* | Vector encoding *pilA* fused to T18 | This work |
| pUT18C-*fimU* | Vector encoding *fimU* fused to T18 | [8] |
| pUT18C-*pilV* | Vector encoding *pilV* fused to T18 | This work |
| pUT18C-*pilW* | Vector encoding *pilW* fused to T18 | This work |
| pUT18C-*pilX* | Vector encoding *pilX* fused to T18 | This work |
| pUT18C-*pilE* | Vector encoding *pilE* fused to T18 | [9] |
| ***E. coli* strains** |  |  |
| DH5α | *F^-^φ80lacZΔM15 Δ(lacZYA-argF)U169 recA1 endA1 hsdR17(rk^−^, mk^+^) phoA supE44 thi-1 gyrA96 relA1 λ−* | Invitrogen |
| SM10 | thi-1 thr leu tonA lacY supE recA::RP4-2-Tc::Mu (Km^R^) | Invitrogen |
| OP50 | Uracil auxotroph, C. elegans food source | [10] |
| BTH 101 | Bacterial two-hybrid reporter strain | Euromedex |
| ***P. aeruginosa* strains** |  |  |
| PAO1 | WT | [11] |
| PAO1 *pilA* | ISphoA/hah transposon insertion at position 163 | [11] |
| PAO1 *fimU* | ISlacZ/hah transposon insertion at position 237 | [11] |
| PAO1 *pilV* | ISphoA/hah transposon insertion at position 122 | [11] |
| PAO1 *pilW* | ISlacZ/hah transposon insertion at position 381 | [11] |
| PAO1 *pilX* | ISphoA/hah transposon insertion at position 182 | [11] |
| PAO1 *pilY1* | ISlacZ/hah transposon insertion at position 1407 | [11] |
| PAO1 *pilE* | ISphoA/hah transposon insertion at position 183 | [11] |
| PAO1 *sadC* | Deletion of *sadC* | This work |
| PAO1 *fimS* | Deletion of *fimS* | This work |
| PAO1 *algR* | Deletion of *algR* | This work |
| PAO1 *algR*_D54A_ | PAO1 expressing the phospho-inactive form of *algR* | This work |
| PAO1 *algR*_D54E_ | PAO1 expressing the phospho-mimetic form of *algR* | This work |
| PA14 | WT | [12] |
| PA14 + pBADGr | WT with pBADGr | This work |
| PA14 + pMS402-P*fimU* | WT with pMS402 containing *fimU* promoter | This work |
| PA14 + pMS402-P*fimU* + pBADGr | WT with pMS402 containing *fimU* promoter and pBADGr | This work |
| PA14 + pMS402-P*cdrA* | WT with pMS402 containing *cdrA* promoter | This work |
| PA14 + pMS402-P*cdrA* + pBADGr | WT with pMS402 containing *cdrA* promoter and pBADGr | This work |
| PA14 *pilA* | Deletion of *pilA* | This work |
| PA14 *pilA* + pMS402-P*fimU* | Deletion of *pilA* with pMS402 containing *fimU* promoter | This work |
| PA14 *pilA* + pMS402-P*cdrA* | Deletion of *pilA* with pMS402 containing *cdrA* promoter | This work |
| PA14 *fimU* | Deletion of *fimU* | This work |
| PA14 *fimU* + pMS402-P*fimU* | Deletion of *fimU* with pMS402 containing *fimU* promoter | This work |
| PA14 *fimU* + pMS402-P*cdrA* | Deletion of *fimU* with pMS402 containing *cdrA* promoter | This work |
| PA14 *pilV* | Deletion of *pilV* | This work |
| PA14 *pilV* + pMS402-P*fimU* | Deletion of *pilV* with pMS402 containing *fimU* promoter | This work |
| PA14 *pilV* + pMS402-P*fimU* + pBADGr | Deletion of *pilV* with pMS402 containing *fimU* promoter and pBADGr | This work |
| PA14 *pilV* + pMS402-P*fimU* + pBADGr-*pilV* | Deletion of *pilV* with pMS402 containing *fimU* promoter and complemented with *pilV* | This work |
| PA14 *pilV* + pMS402-P*cdrA* | Deletion of *pilV* with pMS402 containing *cdrA* promoter | This work |
| PA14 *pilW* | Deletion of *pilW* | This work |
| PA14 *pilW* + pBADGr | Deletion of *pilW* containing pBADGr | This work |
| PA14 *pilW* + pBADGr-*pilW* | Deletion of *pilW* complemented with *pilW* | This work |
| PA14 *pilW* + pMS402-P*fimU* | Deletion of *pilW* with pMS402 containing *fimU* promoter | This work |
| PA14 *pilW* + pMS402-P*fimU* + pBADGr | Deletion of *pilW* with pMS402 containing *fimU* promoter and pBADGr | This work |
| PA14 *pilW* + pMS402-P*fimU* + pBADGr-*pilW* | Deletion of *pilW* with pMS402 containing *fimU* promoter and complemented with *pilW* | This work |
| PA14 *pilW* + pMS402-P*fimU* + pBADGr-*pilY1* | Deletion of *pilW* with pMS402 containing *fimU* promoter and complemented with *pilY1* | This work |
| PA14 *pilW* + pMS402-P*cdrA* | Deletion of *pilW* with pMS402 containing *cdrA* promoter | This work |
| PA14 *pilX* | Deletion of *pilX* | This work |
| PA14 *pilX* + pBADGr | Deletion of *pilX* containing pBADGr | This work |
| PA14 *pilX* + pBADGr-*pilX* | Deletion of *pilX* complemented with *pilX* | This work |
| PA14 *pilX* + pMS402-P*fimU* | Deletion of *pilX* with pMS402 containing *fimU* promoter | This work |
| PA14 *pilX* + pMS402-P*fimU* + pBADGr | Deletion of *pilX* with pMS402 containing *fimU* promoter and pBADGr | This work |
| PA14 *pilX* + pMS402-P*fimU* + pBADGr-*pilX* | Deletion of *pilX* with pMS402 containing *fimU* promoter and complemented with *pilX* | This work |
| PA14 *pilX* + pMS402-P*fimU* + pBADGr-*pilY1* | Deletion of *pilX* with pMS402 containing *fimU* promoter and complemented with *pilY1* | This work |
| PA14 *pilX* + pMS402-P*cdrA* | Deletion of *pilX* with pMS402 containing *cdrA* promoter | This work |
| PA14 *pilY1* | Deletion of *pilY1* | This work |
| PA14 *pilY1* + pBADGr | Deletion of *pilY1* containing pBADGr | This work |
| PA14 *pilY1* + pBADGr-*pilY1* | Deletion of *pilY1* complemented with *pilY1* | This work |
| PA14 *pilY1* + pMS402-P*fimU* | Deletion of *pilY1* with pMS402 containing *fimU* promoter | This work |
| PA14 *pilY1* + pMS402-P*fimU* + pBADGr | Deletion of *pilY1* with pMS402 containing *fimU* promoter and pBADGr | This work |
| PA14 *pilY1* + pMS402-P*fimU* + pBADGr-*pilY1* | Deletion of *pilY1* with pMS402 containing *fimU* promoter and complemented with *pilY1* | This work |
| PA14 *pilY1* + pMS402-P*fimU* + pBADGr-*fimU* | Deletion of *pilY1* with pMS402 containing *fimU* promoter and complemented with *fimU* | This work |
| PA14 *pilY1* + pMS402-P*fimU* + pBADGr-*pilV* | Deletion of *pilY1* with pMS402 containing *fimU* promoter and complemented with *pilV* | This work |
| PA14 *pilY1* + pMS402-P*fimU* + pBADGr-*pilW* | Deletion of *pilY1* with pMS402 containing *fimU* promoter and complemented with *pilW* | This work |
| PA14 *pilY1* + pMS402-P*fimU* + pBADGr-*pilX* | Deletion of *pilY1* with pMS402 containing *fimU* promoter and complemented with *pilX* | This work |
| PA14 *pilY1* + pMS402-P*fimU* + pBADGr-*pilE* | Deletion of *pilY1* with pMS402 containing *fimU* promoter and complemented with *pilE* | This work |
| PA14 *pilY1* + pMS402-P*cdrA* | Deletion of *pilY1* with pMS402 containing *cdrA* promoter | This work |
| PA14 *pilE* | Deletion of *pilE* | This work |
| PA14 *pilE* + pMS402-P*fimU* | Deletion of *pilE* with pMS402 containing *fimU* promoter | This work |
| PA14 *pilE* + pMS402-P*cdrA* | Deletion of *pilE* with pMS402 containing *cdrA* promoter | This work |
| PA14 *sadC* *roeA* | Deletion of *sadC* and *roeA* | [13] |
| PA14 *sadC* | Deletion of *sadC* | This work |
| PA14 *sadC* + pBADGr | Deletion of *sadC* with pBADGr | This work |
| PA14 *sadC* + pMS402-P*cdrA* + pBADGr | Deletion of *sadC* with pMS402 containing *cdrA* promoter and pBADGr | This work |
| PA14 *sadC* + pBADGr-*sadC* | Deletion of *sadC* complemented with *sadC* | This work |
| PA14 *sadC* + pMS402-P*cdrA* + pBADGr-*sadC* | Deletion of *sadC* with pMS402 containing *cdrA* promoter and complemented with *sadC* | This work |
| PA14 *fimS* | Deletion of *fimS* | This work |
| PA14 *fimS* + pMS402-P*fimU* | Deletion of *fimS* with pMS402 containing *fimU* promoter | This work |
| PA14 *algR* | Deletion of *algR* | This work |
| PA14 *algR* + pBADGr | Deletion of *algR* with pBADGr | This work |
| PA14 *algR* + pMS402-P*cdrA* + pBADGr | Deletion of *algR* with pMS402 containing *cdrA* promoter and PA14 | This work |
| PA14 *algR* + pBADGr-*algR* | Deletion of *algR* complemented with WT *algR* | This work |
| PA14 *algR* + pMS402-P*cdrA* + pBADGr-*algR* | Deletion of *algR* with pMS402 containing *cdrA* promoter and complemented with *algR* | This work |
| PA14 *algR* + pBADGr-*algR*_D54A_ | Deletion of *algR* complemented with phospho-inactive *algR* | This work |
| PA14 *algR* + pMS402-P*fimU* | Deletion of *algR* with pMS402 containing *fimU* promoter | This work |
| PA14 *algR*_D54A_ | PA14 expressing the phospho-inactive form of *algR* | This work |
| PA14 *algR*_D54E_ | PA14 expressing the phospho-mimetic form of *algR* | This work |
| PA14 *algU* | Deletion of *algU* | This work |
| PA14 *pilD* | Deletion of *pilD* | This work |
| PA14 *pilW* *fimS* | Deletion of *fimS* in *pilW* background | This work |
| PA14 *pilW* *algR* | Deletion of *algR* in *pilW* background | This work |
| PA14 *pilW* *algR*_D54A_ | Deletion of *pilW* in phospho-inactive *algR* background | This work |
| PA14 *pilW* *algU* | Deletion of *algU* in *pilW* background | This work |
| PA14 *pilX* *fimS* | Deletion of *fimS* in *pilX* background | This work |
| PA14 *pilX* *algR* | Deletion of *algR* in *pilX* background | This work |
| PA14 *pilX* *algR*_D54A_ | Deletion of *pilX* deletion in phospho-inactive *algR* background | This work |
| PA14 *pilX* *algU* | Deletion of *algU* in *pilX* background | This work |
| PA14 *pilY1* *fimS* | Deletion of *fimS* in *pilY1* background | This work |
| PA14 *pilY1* *fimS* + pMS402-P*fimU* | Deletion of *pilY1*/*fimS* with pMS402 containing *fimU* promoter | This work |
| PA14 *pilY1* *algR* | Deletion of *algR* in *pilY1* background | This work |
| PA14 *pilY1* *algR* + pMS402-P*fimU* | Deletion of *pilY1*/*algR* with pMS402 containing *fimU* promoter | This work |
| PA14 *pilY1* *algR*_D54A_ | Deletion of *pilY1* in phospho-inactive *algR* background | This work |
| PA14 *pilY1* *algU* | Deletion of *algU* in *pilY1* background | This work |

**References**

1. Hoang TT, Karkhoff-Schweizer RR, Kutchma AJ, Schweizer HP. A broad-host-range Flp-FRT recombination system for site-specific excision of chromosomally-located DNA sequences: application for isolation of unmarked *Pseudomonas aeruginosa* mutants. Gene. 1998;212(1):77-86. doi: <https://doi.org/10.1016/S0378-1119(98)00130-9>.

2. Asikyan ML, Kus JV, Burrows LL. Novel proteins that modulate type IV pilus retraction dynamics in *Pseudomonas aeruginosa*. J Bacteriol. 2008;190(21):7022-34. Epub 2008/09/09. doi: 10.1128/jb.00938-08. PubMed PMID: 18776014; PubMed Central PMCID: PMCPMC2580705.

3. Giltner CL, Rana N, Lunardo MN, Hussain AQ, Burrows LL. Evolutionary and functional diversity of the *Pseudomonas type* IVa pilin island. Environ Microbiol. 2011;13(1):250-64. doi: 10.1111/j.1462-2920.2010.02327.x.

4. Mulcahy H, Charron-Mazenod L, Lewenza S. Extracellular DNA chelates cations and induces antibiotic resistance in *Pseudomonas aeruginosa* biofilms. PLoS Pathog. 2008;4(11):e1000213. doi: 10.1371/journal.ppat.1000213. PubMed PMID: PMC2581603.

5. Karimova G, Pidoux J, Ullmann A, Ladant D. A bacterial two-hybrid system based on a reconstituted signal transduction pathway. Proc Natl Acad Sci U S A. 1998;95(10):5752-6. Epub 1998/05/20. PubMed PMID: 9576956; PubMed Central PMCID: PMCPMC20451.

6. Karimova G, Ullmann A, Ladant D. Protein-protein interaction between *Bacillus stearothermophilus* tyrosyl-tRNA synthetase subdomains revealed by a bacterial two-hybrid system. J Mol Microbiol Biotechnol. 2001;3(1):73-82. Epub 2001/02/24. PubMed PMID: 11200232.

7. Kilmury SLN, Burrows LL. Type IV pilins regulate their own expression via direct intramembrane interactions with the sensor kinase PilS. Proc Natl Acad Sci U S A. 2016;113(21):6017-22. doi: 10.1073/pnas.1512947113.

8. Nguyen Y, Sugiman-Marangos S, Harvey H, Bell SD, Charlton CL, Junop MS, et al. *Pseudomonas aeruginosa* minor pilins prime type IVa pilus assembly and promote surface display of the PilY1 adhesin. J Biol Chem. 2015;290(1):601-11. doi: 10.1074/jbc.M114.616904. PubMed PMID: PMC4281761.

9. Nguyen Y, Harvey H, Sugiman-Marangos S, Bell SD, Buensuceso RNC, Junop MS, et al. Structural and functional studies of the *Pseudomonas aeruginosa* minor pilin, PilE. J Biol Chem. 2015;290(44):26856-65. doi: 10.1074/jbc.M115.683334.

10. Brenner S. The genetics of *Caenorhabditis elegans*. Genetics. 1974;77(1):71-94. Epub 1974/05/01. PubMed PMID: 4366476; PubMed Central PMCID: PMCPMC1213120.

11. Jacobs MA, Alwood A, Thaipisuttikul I, Spencer D, Haugen E, Ernst S, et al. Comprehensive transposon mutant library of *Pseudomonas aeruginosa*. Proc Natl Acad Sci U S A. 2003;100(24):14339-44. doi: 10.1073/pnas.2036282100.

12. Rahme LG, Stevens EJ, Wolfort SF, Shao J, Tompkins RG, Ausubel FM. Common virulence factors for bacterial pathogenicity in plants and animals. Science. 1995;268(5219):1899-902. Epub 1995/06/30. PubMed PMID: 7604262.

13. Merritt JH, Brothers KM, Kuchma SL, O'Toole GA. SadC reciprocally influences biofilm formation and swarming motility via modulation of exopolysaccharide production and flagellar function. J Bacteriol. 2007;189(22):8154-64. Epub 2007/06/26. doi: 10.1128/jb.00585-07. PubMed PMID: 17586642; PubMed Central PMCID: PMCPMC2168701.
