# Supplementary material for: Pseudomonas aeruginosa type IV minor pilins and PilY1 regulate virulence by modulating FimS-AlgR activity
Source: PLoS Pathog. 2018 May 18;14(5):e1007074. doi: 10.1371/journal.ppat.1007074 (PMC5979040; doi:10.1371/journal.ppat.1007074)
Supplement: S2 Table — Restriction sites are underlined. (DOCX) [file ppat.1007074.s008.docx]

**Table S2. Primers used in this study**. Restriction sites are underlined.

| **Primer name** | **Sequence** |
| --- | --- |
| pilA(-1100)F | TCGAGGATCCGCATCACGATCTTCTC |
| pilA(52)R | TACCTGCAGTCGCAACCACGATCATCAG |
| pilA(62)F | ACCTGCAGATATGCCTGCCCTGACTGCA |
| pilA(+1056)R | CTGGAAGCTTCCGGCGGAATCAACG |
| pilX CF | GTCGAATTCATGACCCTGCGCCATACCTCTC |
| pilX CR | GACAAGCTTTCAGTTGGTATACAGGCGTGCA |
| pilY1 CF | GTCAGAATTCTGGAGCCAGCGCATGATC |
| pilY1 CR | CTATCCCGGGTCATTTCTCCTCGACGAC |
| sadC(-500)F | GATTGAATTCGAGCTCGAACACGGTGACGATCCCG |
| sadC(+558)R | CTAATCTAGAGGATCCCAGTCCGGCTCGTAGCGC |
| algR F1 | GCAGAATTCGGCCGAGCATGCGGTG |
| algR R1 | GCAGGATCCGAGGTTCGTCATCGA |
| algR F2 | GCAGGATCCGCCGGAGTCAGGCG |
| algR R2 | GCAAAGCTTCGCAGGCTGGAGGTG |
| fimS(-500)F | GACTGGTACCGTTCATGTGCACGTCTTCCAG |
| fimS(+500)R | GCCGAAGCTTTGTGGTCGGCAATGAAGAAG |
| fimS(18)F | GTACAACCATGGTAAGTTCCTTGAATCGGATAGGC |
| fimS(15)R | GAACTTACCATGGTTGTACATGCAGGAAGCCTGA |
| algRD54-500F | CGGCTCTAGATGAGCAGTATCGTCTTGGCGATCG |
| algRD54+500R | GATTAAGCTTGCACGAAGCGCTCGCCGAAC |
| algR(D54A)F | ATCGTCCTGCTGGCTATCCGCATGCC |
| algR(D54A)R | GGCATGCGGATAGCCAGCAGGACGAT |
| algR(D54E)F | ATCGTCCTGCTGGAAATCCGCATGCCC |
| algR(D54E)R | GGGCATGCGGATTTCCAGCAGGACGAT |
| algU 74R | GACTAGATCTAGACATGTCTGAGCAGATCGAAAGC |
| algU 51F | CAGACATGTCTAGATCTAGTCGCTCGTGAAGCAATC |
| algU -478F | GTGAGCTCTCAAGGCCAGACTCAG |
| algU +499R | GAAAGCTTGGTATCGCTGGACGAGGAG |
| pilD 90R | GACTACTCTAGACGATGGTTGAGGAAGCTGCC |
| pilD 85F | CATCGTCTAGAGTAGTCCTATCTGGCGATTGC |
| pilD -638F | CGGAGCTCCAGTTCCAGTCCGTATTTG |
| pilD +456R | GTAAGCTTCCTGGAGGATCGAGCGC |
| algR CF | GTAACCATGGCTCATGCAGGAAGCCTGAGCTTATG |
| algR CR | CAGTAAGCTTTCAGAGCTGATGCATCAGACGCCTG |
| PfimU F | GTTAGGATCCGCTCTCTTACCTGTGCTCCA |
| PfimU R | GCATGGATCCGCAGTACTCCACAAGGAAAAG |
| PcdrA-500F | GAGGATCCGATCGGCGCCTTGTTGCTG |
| PcdrA-1R | GCGGATCCGAAAATCTCCCTATCTGCGTGGC |
| FimS Bac-F | CATTCTAGACATGCCTATCCGATTCAAG |
| FimS Bac-R | CCTGAATTCTCAGGCTTCCTGCATGAGTCG |
| AlgR BACTH F | GCAGGATCCCATGAATGTCCTGATTGTCG |
| AlgR BACTH R | GCAGGTACCGAGAGCTGATGCATCAGACG |
| pilAB2HFor | GCATCTAGACTTTACCTTGATCGAACTGATGATCGTGGTTG |
| pilA2B2HRev | CATGAATTCTTAGTTATCACAACCTTTCGGAGTGAACATCGG |
| pilVB2HFor | GCATCTAGACTTCAGCATGATCGAAGTGCTGGTCG |
| pilVB2HRev | CATGGTACCTCATGGCTCGACCCTGAGG |
| pilWB2HFor | GCATCTAGACCTGTCCATGATCGAACTACTGGTGGCC |
| pilWMCS2Rev | AAGGTACCTCATGGCACGAGATTCCTGAGTGTCTGG |
| pilXB2HFor | GTATCTAGACGCCACGCTGGTCATCGCC |
| pilXMCS2Rev | AAGGTACCTCAGTTGGTATAGAGACGGGCGAGAA |
