# Supplementary material for: Pseudomonas aeruginosa type IV minor pilins and PilY1 regulate virulence by modulating FimS-AlgR activity
Source: PLoS Pathog. 2018 May 18;14(5):e1007074. doi: 10.1371/journal.ppat.1007074 (PMC5979040; doi:10.1371/journal.ppat.1007074)
Supplement: S1 File — Three independent experiments for Figs 1A, 1B, 3B, 5A, 5B, 6A, 7A–7C and 8, and S1B, S3A, S3B and S5C Figs. (PDF) [file ppat.1007074.s009.pdf]

Figure 1A

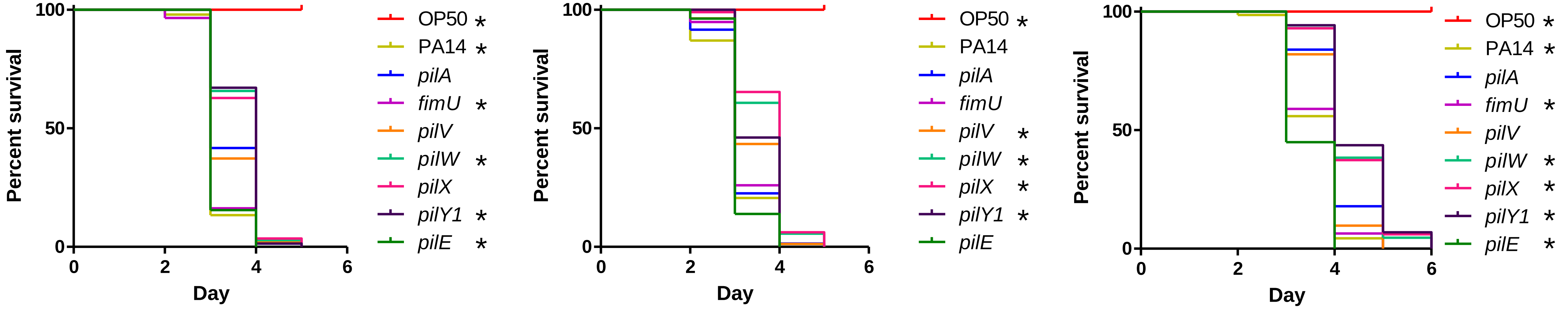

Three trials for Fig 1A. SK assays for PA14 *pilA*, *fimU*, *pilV*, *pilW*, *pilX*, *pilY1*, and *pilE* mutants. PA14 *fimU* and *pilE* mutants had similar virulence to WT, *pilA* and *pilV* mutants were slightly less virulent than WT, and *pilW, pilX,* and *pilY1* mutants were less virulent than all other strains tested in the majority of trials. Asterisks indicate strains that were significantly different from a *pilA* mutant by Gehan-Breslow-Wilcoxon test at $p = 0.05$ ($p = 0.00625$ with a Bonferroni correction).

Figure 1B

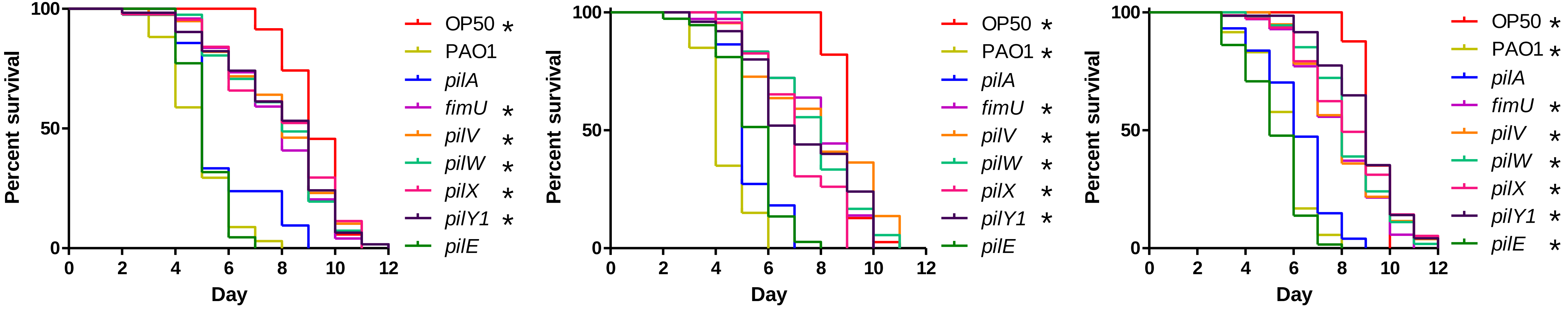

Three trials for Fig 1B. SK assays for PAO1 *pilA*, *fimU*, *pilV*, *pilW*, *pilX*, *pilY1*, and *pilE* mutants. The PAO1 *pilE* mutant had similar virulence to WT, the *pilA* mutant was slightly less virulent, and *fimU*, *pilV*, *pilW*, *pilX*, and *pilY1* mutants were much less virulent. Asterisks indicate strains that were significantly different from a *pilA* mutant by Gehan-Breslow-Wilcoxon test at p = 0.05 (p = 0.00625 with a Bonferroni correction).

Figure 3B

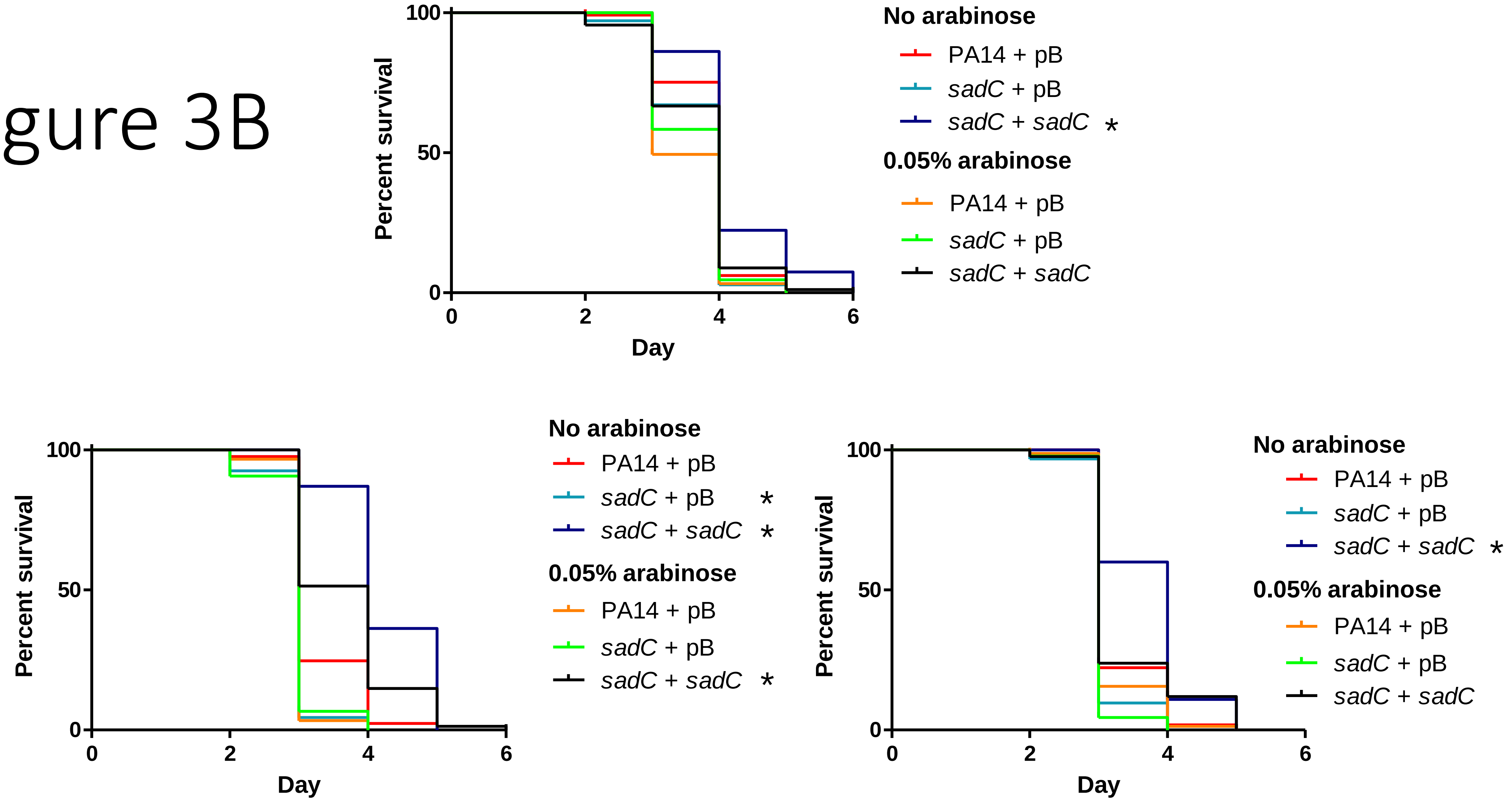

Three trials for Fig 3B. SK assays for *sadC* deletion and overexpression strains. Overexpression of SadC led to a subtle but reproducible loss of virulence relative to WT at 0% arabinose. A *sadC* mutant had WT virulence. Asterisks indicate strains that were significantly different from PA14 + pBADGr by Gehan-Breslow-Wilcoxon test at p = 0.05 (p = 0.0125 with a Bonferroni correction).

Figure 5A

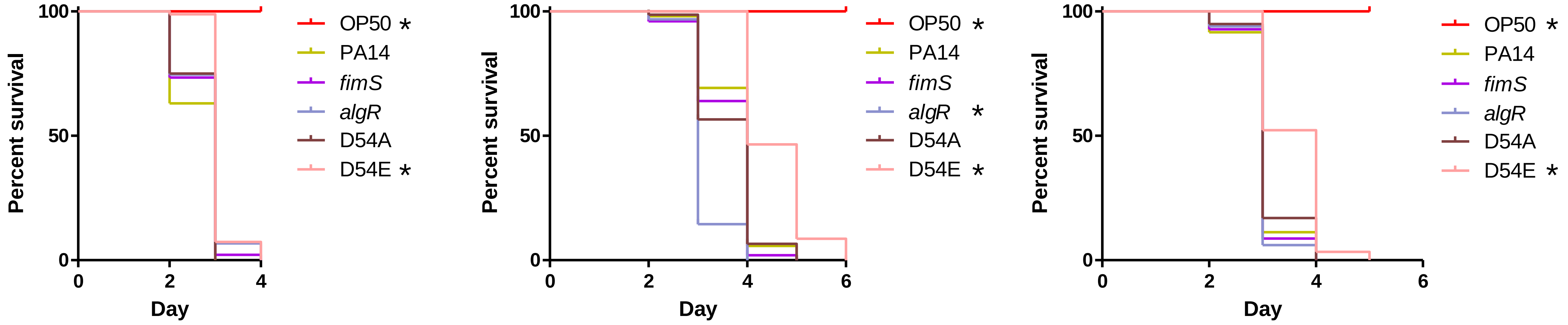

Three trials for Fig 5A. SK assays for PA14 *fimS*, *algR*, *algR*$_{D54A}$, and *algR*$_{D54E}$ mutants. The *fimS*, *algR,* and *algR*$_{D54A}$ mutants had WT virulence, while the *algR*$_{D54E}$ mutant was less virulent than WT. Asterisks indicate strains that were significantly different from WT by Gehan-Breslow-Wilcoxon test at p = 0.05 (p = 0.01 with a Bonferroni correction).

Figure 5B

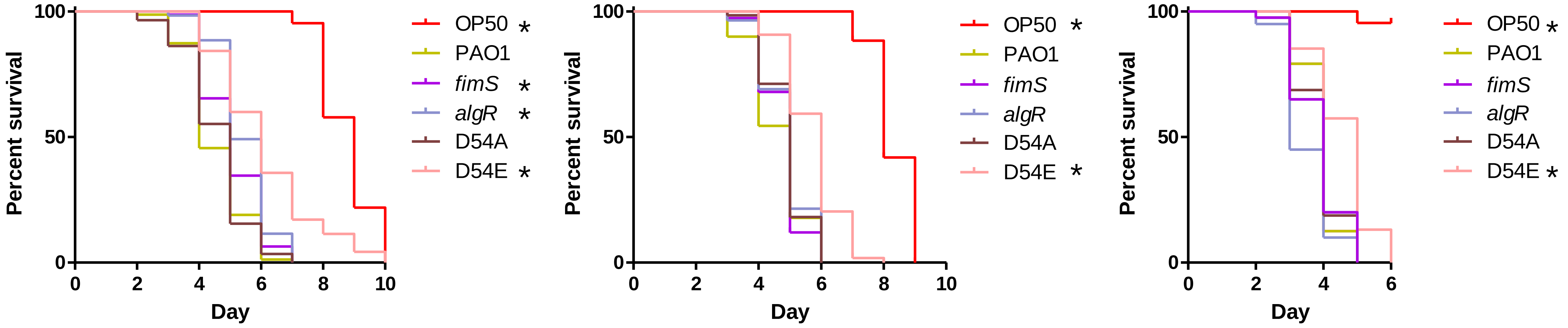

Three trials for Fig 5B. SK assays for PAO1 *fimS*, *algR*, *algR*$_{D54A}$, and *algR*$_{D54E}$ mutants. The *fimS*, *algR,* and *algR*$_{D54A}$ mutants had WT virulence, while the *algR*$_{D54E}$ mutant was less virulent than WT. Asterisks indicate strains that were significantly different from WT by Gehan-Breslow-Wilcoxon test at p = 0.05 (p = 0.01 with a Bonferroni correction).

Figure 6A

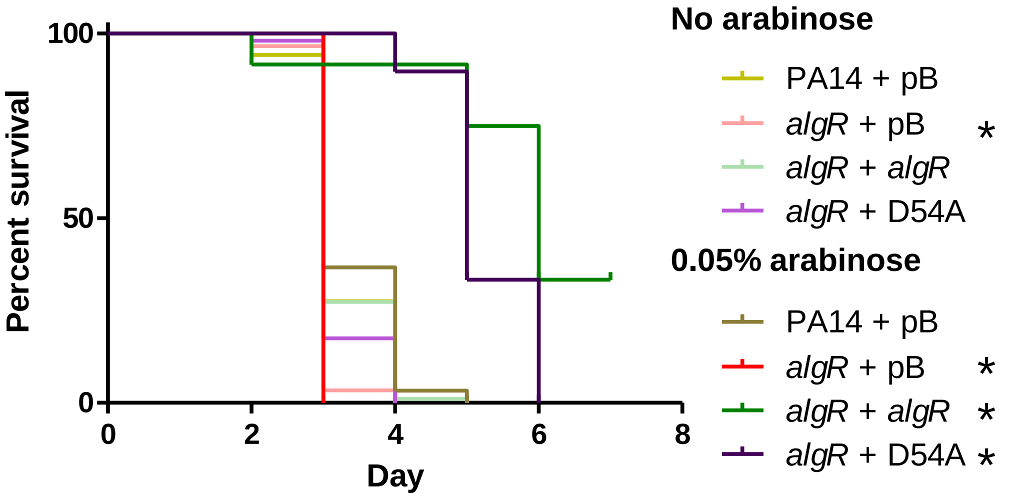

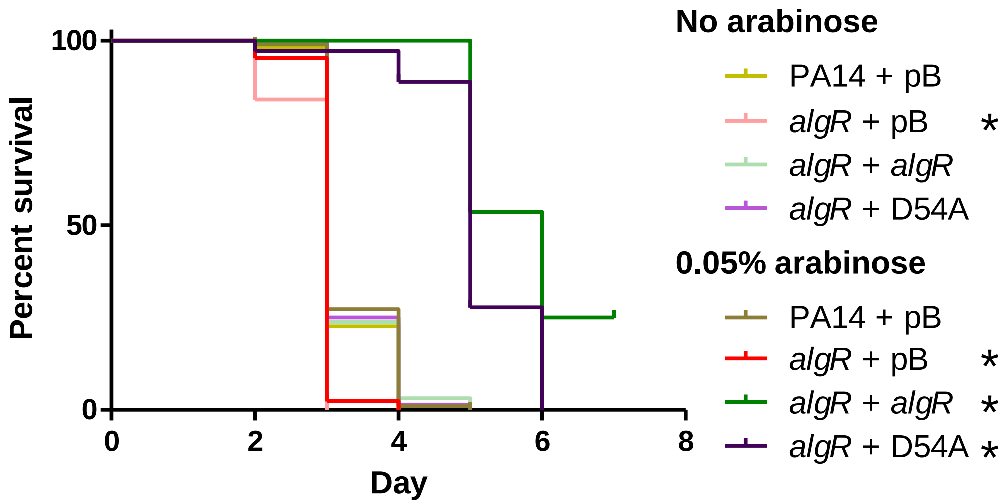

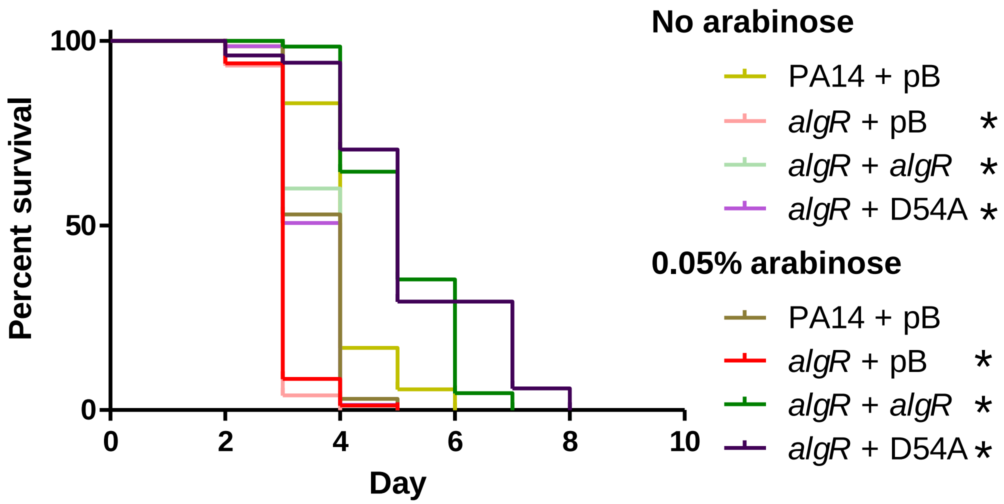

Three trials for Fig 6A. SK assays for *algR* deletion and overexpression strains. Loss of *algR* led to a small increase in virulence, while overexpression of pBADGr-*algR* or pBADGr-*algR*$_{\text{D54A}}$ reduced virulence at 0.05% arabinose. Asterisks indicate strains that were significantly different from PA14 + pBADGr by Gehan-Breslow-Wilcoxon test at p = 0.05 (p = 0.00833 with a Bonferroni correction).

Figure 7A

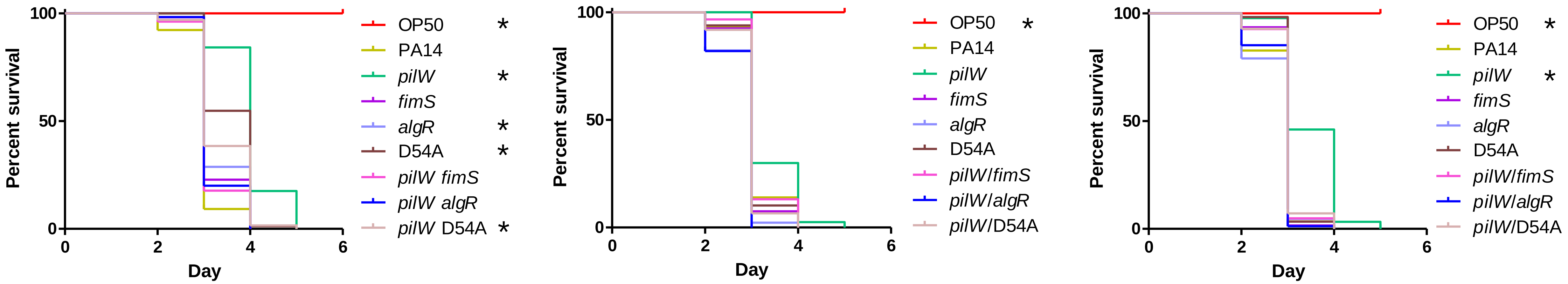

Three trials for Fig 7A. SK assays for *pilW fimS*, *pilW algR*, and *pilW* $algR_{D54A}$ mutants. *fimS*, *algR*, and $algR_{D54A}$ mutants have WT virulence. *pilW* had reduced virulence relative to WT, *fimS*, *algR*, and $algR_{D54A}$ mutants. Combination of *pilW* with *fimS*, *algR*, or $algR_{D54A}$ mutations results in virulence equivalent to *fimS*, *algR*, and $algR_{D54A}$ single mutants, respectively. Asterisks indicate strains that were less virulent than PA14 by Gehan-Breslow-Wilcoxon test at p = 0.05 (p = 0.003125 with a Bonferroni correction).

Figure 7B

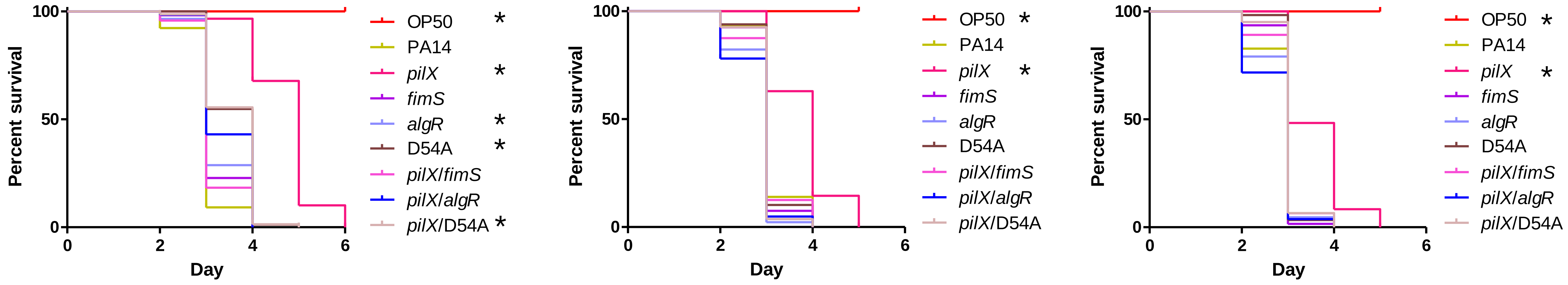

Three trials for Fig 7B. SK assays for *pilX fimS*, *pilX algR*, and *pilX algR*$_{D54A}$ mutants. *fimS*, *algR*, and *algR*$_{D54A}$ mutants have WT virulence. *pilX* had reduced virulence relative to WT, *fimS*, *algR*, and *algR*$_{D54A}$ mutants. Combination of *pilX* with *fimS*, *algR*, or *algR*$_{D54A}$ mutations results in virulence equivalent to *fimS*, *algR*, and *algR*$_{D54A}$ single mutants, respectively. Asterisks indicate strains that were less virulent than PA14 by Gehan-Breslow-Wilcoxon test at $p = 0.05$ ($p = 0.003125$ with a Bonferroni correction).

Figure 7C

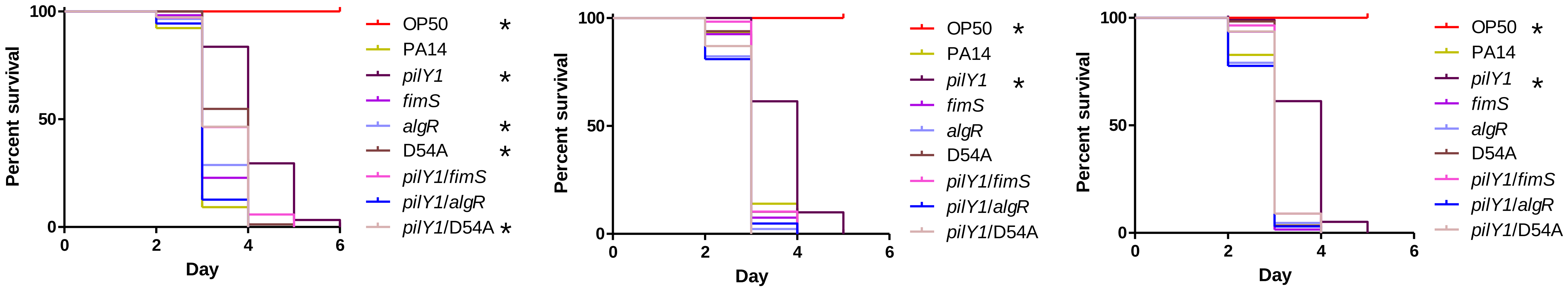

Three trials for Fig 7C. SK assays for *pilY1 fimS*, *pilY1 algR*, and *pilY1 algR*$_{D54A}$ mutants. *fimS*, *algR*, and *algR*$_{D54A}$ mutants have WT virulence. *pilY1* had reduced virulence relative to WT, *fimS*, *algR*, and *algR*$_{D54A}$ mutants. Combination of *pilY1* with *fimS*, *algR*, or *algR*$_{D54A}$ mutations results in virulence equivalent to *fimS*, *algR*, and *algR*$_{D54A}$ single mutants, respectively. Asterisks indicate strains that were less virulent than PA14 by Gehan-Breslow-Wilcoxon test at $p = 0.05$ ($p = 0.003125$ with a Bonferroni correction).

Figure 8

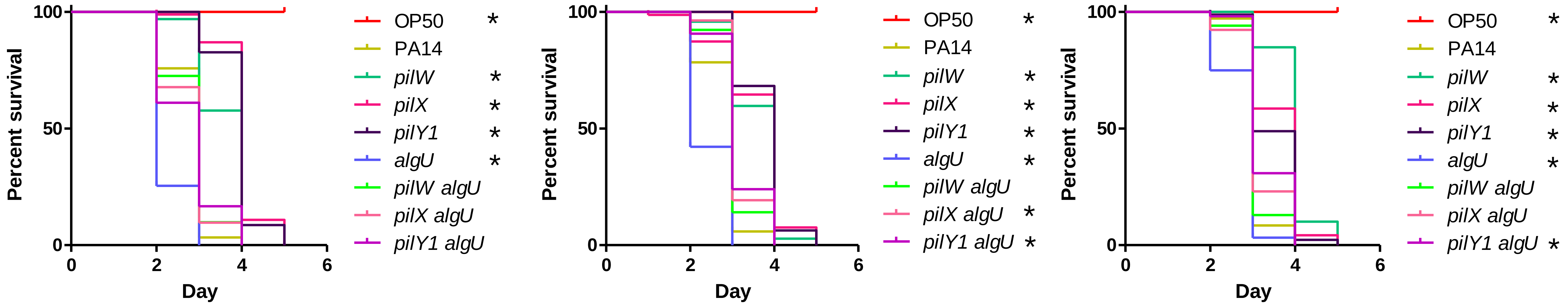

Three trials for Fig 8. SK assays for PA14 *pilW*, *pilX*, *pilY1*, *algU*, *pilW algU*, *pilX algU*, and *pilY1 algU* mutants. Loss of *algU* led to increased pathogenicity relative to WT, while *pilW algU*, *pilX algU*, and *pilY1 algU* mutants had near-WT virulence. Asterisks indicate strains that were less virulent than PA14 by Gehan-Breslow-Wilcoxon test at $p = 0.05$ ($p = 0.00625$ with a Bonferroni correction).

Figure S1B

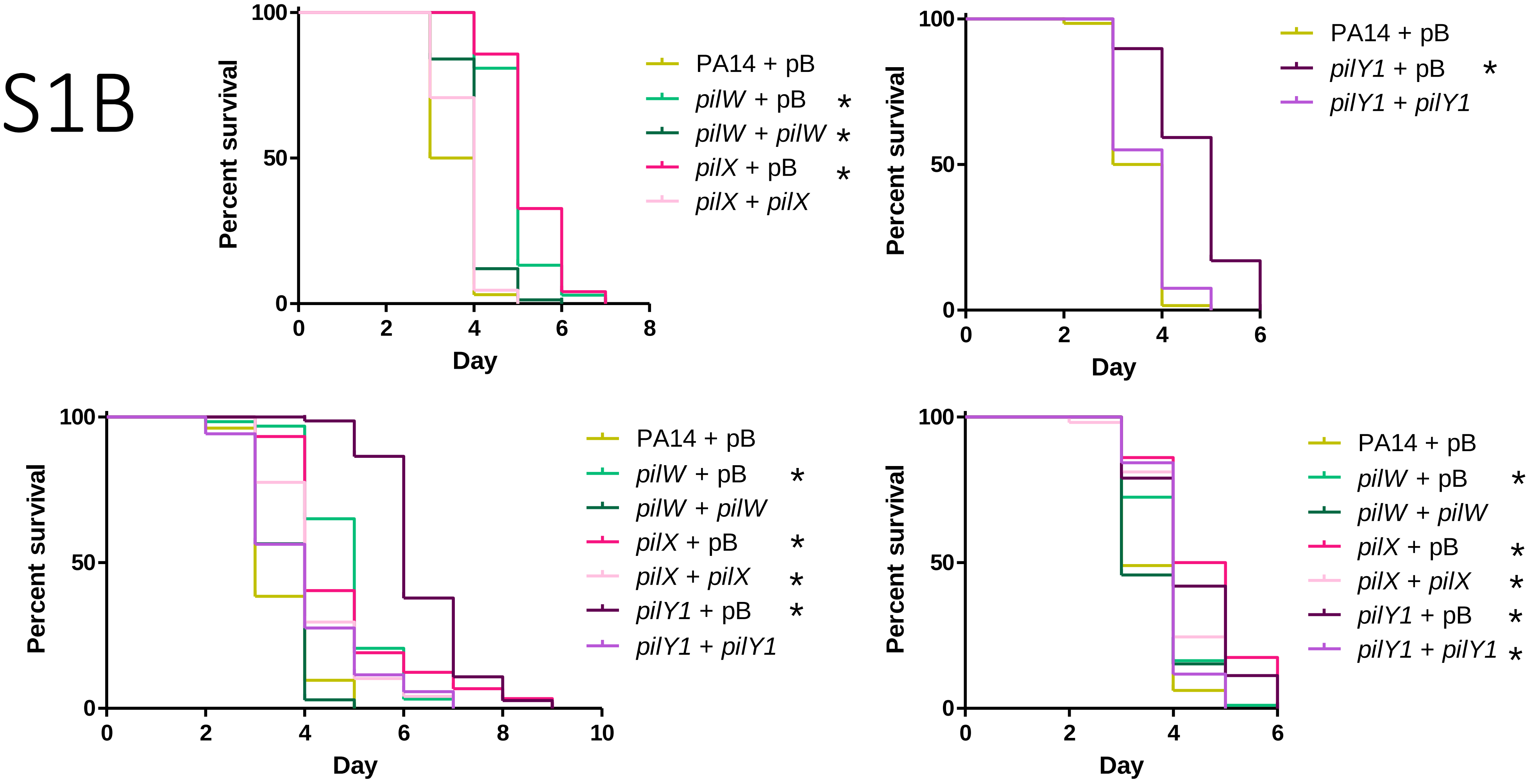

Three trials for Fig S1B. SK assays for complemented PA14 *pilW*, *pilX*, and *pilY1* mutants. Complementation of *pilW*, *pilX*, and *pilY1* mutants with pBADGr-*pilW*, pBADGr-*pilX*, or pBADGr-*pilY1*, respectively, restored virulence to near-WT levels. Asterisks indicate strains that were less virulent than WT by Gehan-Breslow-Wilcoxon test at p = 0.05 (p = 0.00833 with a Bonferroni correction), n = 3.

Figure S3A

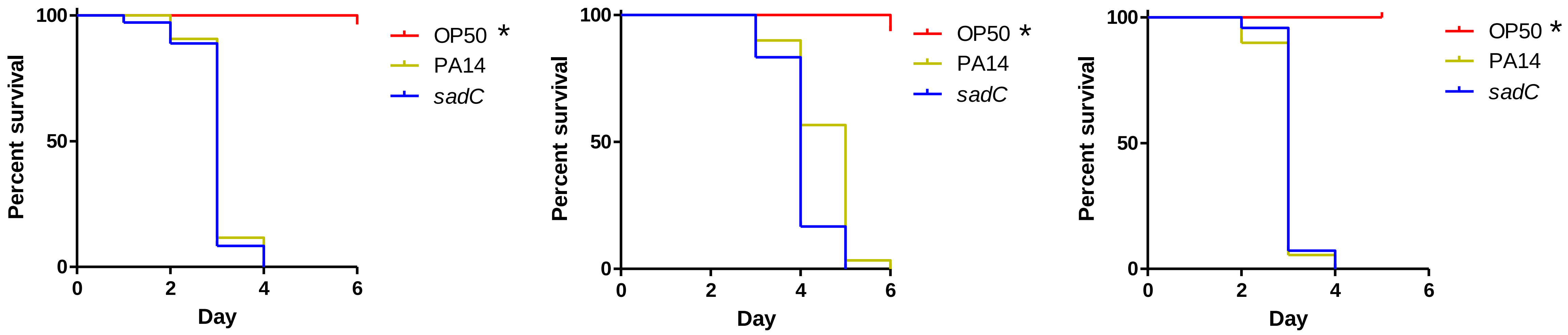

Three trials for Fig S3A. SK assays for PA14 *sadC* mutants. Loss of *sadC* had no impact on pathogenicity relative to WT, as measured by Gehan-Breslow-Wilcoxon test at $p = 0.05$ ($p = 0.025$ with a Bonferroni correction).

Figure S3B

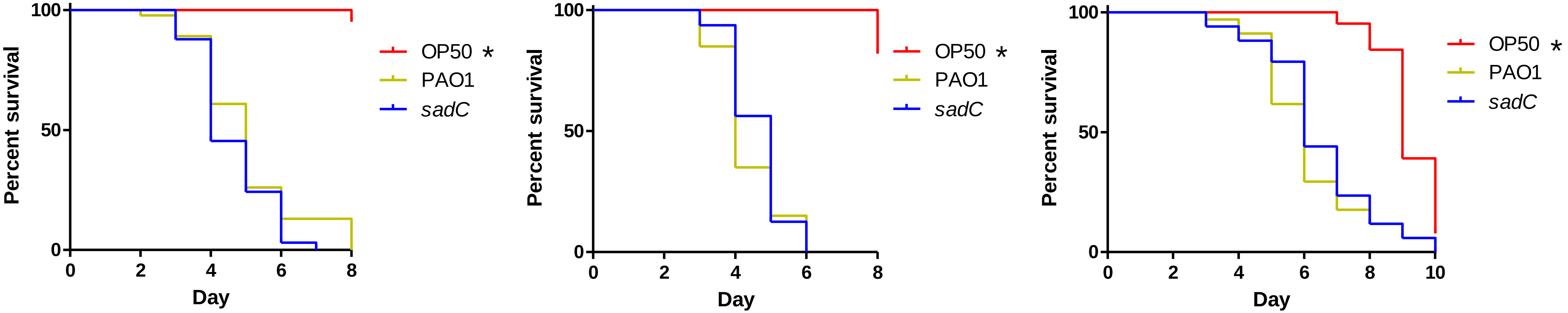

Three trials for Fig S3B. SK assays for PAO1 *sadC* mutants. Loss of *sadC* had no impact on pathogenicity relative to WT, as measured by Gehan-Breslow-Wilcoxon test at p = 0.05 (p = 0.025 with a Bonferroni correction).

Figure S5C

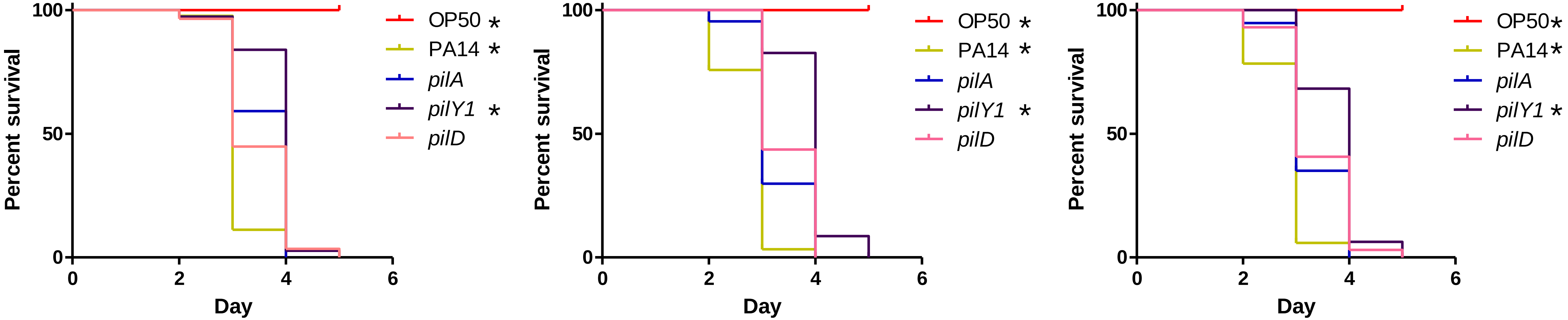

Three trials for Fig S5C. SK assays for PA14, *pilA*, *pilY1*, and *pilD* mutants. A *pilD* mutant had equivalent virulence to a *pilA* mutant; less pathogenic than WT but more pathogenic than a *pilY1* mutants. Asterisks represent strains that were significantly different from the *pilA* mutant by Gehan-Breslow-Wilcoxon test at p = 0.05 (p = 0.0125 with a Bonferroni correction).
